# Active Pneumatic Vibration Control by Using Pressure and Velocity Measurements and Adaptive Fuzzy Sliding-Mode Controller

**DOI:** 10.3390/s130708431

**Published:** 2013-07-02

**Authors:** Hung-Yi Chen, Jin-Wei Liang, Jia-Wei Wu

**Affiliations:** Ming Chi University of Technology, No. 84, Gungjuan Road, Taishan, New Taipei City 243, Taiwan; E-Mails: liangj@mail.mcut.edu.tw (J.-W.L.); m991e8022@mail2.mcut.edu.tw (J.-W.W.)

**Keywords:** pneumatic vibration isolation (PVI) system, adaptive fuzzy sliding-mode controller, online learning ability

## Abstract

This paper presents an intelligent control strategy to overcome nonlinear and time-varying characteristics of a diaphragm-type pneumatic vibration isolator (PVI) system. By combining an adaptive rule with fuzzy and sliding-mode control, the method has online learning ability when it faces the system's nonlinear and time-varying behaviors during an active vibration control process. Since the proposed scheme has a simple structure, it is easy to implement. To validate the proposed scheme, a composite control which adopts both chamber pressure and payload velocity as feedback signal is implemented. During experimental investigations, sinusoidal excitation at resonance and random-like signal are input on a floor base to simulate ground vibration. Performances obtained from the proposed scheme are compared with those obtained from passive system and PID scheme to illustrate the effectiveness of the proposed intelligent control.

## 1. Introduction

Precision instruments such as laser interferometers, electron-beam microscopes and many others applied in the processing of semiconductors are highly sensitive to ground or environmental vibrations. Since high precision is needed at the presence of these apparatus, requirements on the ground vibration level have become more stringent in regulations or standards [1,2]. As a solution, pneumatic vibration isolators (PVIs) are often used to isolate vibration from the ground or environment. PVIs have found many applications in precision industry, especially in those operating at low-frequency range or supporting heavy static payloads with small dynamic loadings. A pneumatic isolation table system is usually supported by several PVIs which can be actively controlled by servo valves to attenuate vibration transmitted from the floor and suppress any vibration of the table itself. PVI table systems can usually offer good performances in frequency range fairly above the system's natural frequencies, which are typically 2–6 Hz [3]. Nevertheless, the performance deteriorates when excitation frequency gets close to the system's natural frequency.

Due to the compressibility of air and other nonlinearity associated with servo valves, active control of pneumatic pressure has long been known as a challenging task. Little research exists focusing on active pressure control of pneumatic vibration isolators pertaining ground excitations. Among them, Kato *et al.* [4] investigated a pneumatic isolation table using a pressure differentiator and a spool-type servo valve. Although the experimental results reported in [4] demonstrated efficient isolation performance with less energy compared to conventional systems that use nozzle-flapper type servo valves, the approach required detailed modeling of the pressure differentiator. On the other hand, Shin and Kim [3] employed time delay control (TDC) to enhance the low-frequency performance of a pneumatic isolator. Effectiveness of the TDC approach was demonstrated. The method, however, requires a mathematical model of the PVI. Chang *et al.* [5] proposed a new state space model of the PVI that utilized an input-output linearization technique. Based on this new model, a TDC method was designed and verified with experiments on a single chamber PVI. This study also required modeling knowledge of the system.

Dynamics of the PVI are complicated, thus, establishing an appropriate dynamic model for a model-based controller design is not trivial. To this end, a model-free fuzzy control strategy seems preferable. Since traditional a fuzzy logic controller (FLC) needs only simple computation and programming to mimic human control behavior, it has been widely employed in engineering applications. The method, however, has no analytical tool to ensure the stability of closed-loop systems. Moreover, the design of a traditional fuzzy controller depends immensely on an expert, or the experience of an operator in establishing the fuzzy rule bank. Knowledge of the latter is generally difficult to obtain. Thus, a time-consuming adjustment process is required if a specified control performance is to be achieved. In order to improve traditional fuzzy control, Hsiao et *al.* [6,7] proposed a Takagi-Sugeno (T-S) fuzzy controller approach to deal with nonlinear systems. Hsu *et al.* [8] proposed a dynamic TSK-type RBF-based neural-fuzzy controller to control a chaotic system and an inverted pendulum. The stability of the controller is proved in the Lyapunov sense. Chen [9,10] and Chen *et al.* [11] designed the controller and provided the stability conditions for a class of structural and mechanical system represented by Takagi-Sugeno (T-S) fuzzy models and linear matrix inequality (LMI) theory. Additionally, sliding-mode control was introduced into the fuzzy controller so that stability analysis can be carried out using the resultant control scheme. For example, Wang [12] employed a switch function as the fuzzy input variable and proposed a fuzzy sliding-mode controller. Lu and Chen [13] applied similar idea to develop a self-organizing fuzzy sliding-mode controller which solved chattering phenomenon. These approaches, however, depend on a certain model in designing the sliding-mode controller. The fuzzy rule bank design and related calculation adopted in these approaches are still complicated. In contrast, an adaptive fuzzy sliding-mode controller (AFSMC) was proposed by Huang *et al.* [14,15] to monitor the alloying process temperature and suppress vehicle vibration. Since this AFSMC approach has learning ability, it can continuously establish and regulate the fuzzy rule bank and parameters. The associated control implementation can be started with zero initial fuzzy rules. Moreover, its control rules can be reduced to as few as seven rules for various operations. Hence, this approach can significantly reduce the database burden, computational time and expert experience dependence. In this paper, AFSMC controllers are adopted to handle PVI table system control. Control performances are evaluated based on experimental results. During our experimental investigations, a composite control scheme using both chamber pressure and payload velocity measurements as feedback signals will be implemented.

## 2. Experimental Set-up

**Figure 1.**
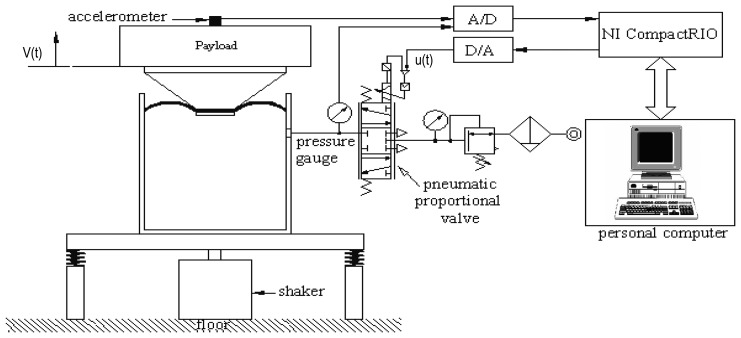
The schematic diagram of the experimental set up.

The pneumatic vibration isolation (PVI) system adopted in this paper consists of a single pneumatic chamber, a rubber diaphragm and a piston that supports the payload as shown in Figure 1. The payload mass is 42 kg. The PVI is used as an actuator in the active-isolation cases. The design of the PVI resembles that of a commercial product (FAEBI-HD series, Bilz Corporation).

**Figure 2.**
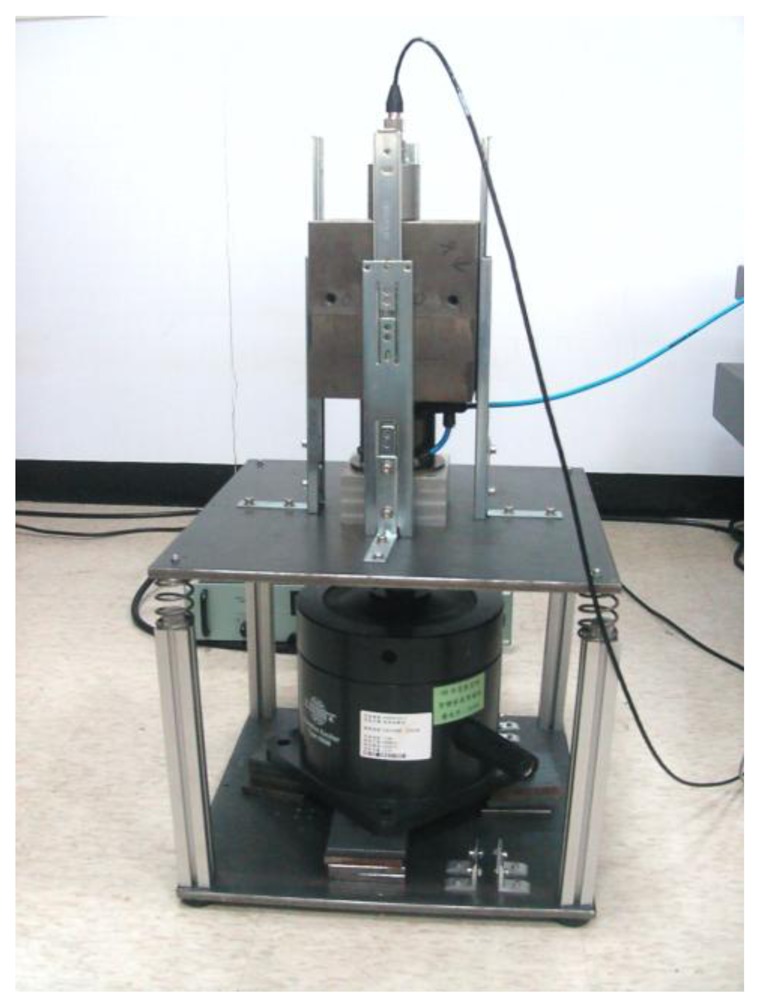
Photograph of the experimental set-up.

This specific design has a relatively small load volume and a large damping volume. It turns out that the total volume of the chamber is about 2.0 × 1CT^−4^ m^3^ while the effective piston area is about 1.96 x 10 ^−3^ m^2^. In order to measure the isolation performances, an accelerometer is installed on the top of the payload. In addition, an electromagnetic shaker is used to excite the floor base so as to simulate the ground vibration input. Both chamber pressure and payload velocity measurements are adopted as the feedback signals in closed-loop control design. Here, the velocity signal was obtained by numerically integrating the acceleration signal. The latter was sensed by a sensitive accelerometer (B&K 8340 with a sensitivity of 9,237 mV/g) mounted on the payload. On the other hand, the pressure signal was measured by a pressure sensor (FESTO SDE1-D6-G2-W18-C-NU-M8 with an accuracy of 2% of final value,) mounted in between the exit of the servo valve and the air chamber. The pressure sensor is so located that the pressure measurement equivalently reveals the pressure dynamics of the air chamber. Figure 2 presents a photograph of the experimental set-up.

The PC-based control unit, using NI-CompactRIO (NI CRIO-9004) and Lab VIEW software, takes measurements through A/D conversions, computes the required control input, and transmits the control input in the form of analog voltage to the pneumatic control valve. The pneumatic control valve, also called servo valve, is a proportional directional control valve (FESTO, MPYE-5-M5-010-B) which generates air mass-flow in proportion to the control voltage sent from the control unit. The spool-type control valve works not only to maintain a static pressure but also supply the required dynamic pressure. To avoid pipe loss, the distance between the control servo valve and pneumatic chamber is kept as small as possible.

During the experimental study, control performances obtained from active-isolation algorithms are compared with those obtained from the passive isolation and PID control scheme. The passive isolation can be accomplished by keeping a static pressure in the PVI where the passive-isolation effects were provided by the rubber diaphragm and the orifice flow through inlet and outlet of the directional control valve. No power input is needed in this case. In contrast with the passive case, voltage input was continuously calculated in accordance with active-control algorithms in the active-isolation cases. Control voltage computed was sent to control the spool motion of the directional control valve so that vibration disturbances of the system can be counteracted and the payload can be kept in a static position.

## 3. Controller Design

In this section, control algorithms applied to deal with the nonlinear pneumatic system are elaborated. The fundamental idea here is to capture nonlinear time-varying system dynamics by using the adaptive fuzzy sliding-mode controller (AFSMC). Although it is unnecessary to estimate the nominal system model in the design of the proposed AFSMC scheme, the description of applicable systems should be clarified as in the followings which also introduce the sliding dynamics to describe the desired system behavior and to represent the objective of the learning controller. Also, in general, it is not easy to measure all the state variables and design a model-based controller for the objective of disturbance rejection in PVI systems. Hence, the sliding-mode concept is combined with fuzzy control strategy here to form a model-free AFSMC for the nonlinear diaphragm-type PVI system control. This simple adaptive fuzzy sliding-mode controller was designed with only 1D fuzzy rule together with two state variables. To briefly introduce the idea of the AFSMC, consider the following autonomous nonlinear uncertain system whose state-space model can be represented as a companion form:

(1) $$\begin{array}{c} {\dot{x}}_{1}\left(t\right)=x_{2}\left(t\right)\\ {\dot{x}}_{2}\left(t\right)=x_{3}\left(t\right)\\ \vdots \\ {\dot{x}}_{n}\left(t\right)=f\left(\text{X}\right)+b\left(\text{X}\right)u\left(t\right)+d\left(t\right) \end{array}$$

where the state vector is **X***^T^* =[*x*_1_, *x*_2_, …*.x_n_*], *u*(*t*) is the control input while *d*(*t*) represents the external disturbances. *f*(**X**) is a function of state variables representing the system dynamics. If the errors of state variables are defined as *e_i_* = *x_id_*− *x_i_*, Equation (1) can be arranged as follows:

(2) $$\begin{array}{c} {\dot{e}}_{1}\left(t\right)=e_{2}\left(t\right)\\ {\dot{e}}_{2}\left(t\right)=e_{3}\left(t\right)\\ \vdots \\ {\dot{e}}_{n}\left(t\right)={\dot{x}}_{nd}\left(t\right)-f\left(\text{X}\right)-b\left(\text{X}\right)u\left(t\right)-d\left(t\right) \end{array}$$

According to the sliding-mode control theory, if all the terms appeared in Equation (2) are known or can be measured, then a perfect control law based on feedback linearization can be obtained as:

(3) $$u_{eq}\left(t\right)=\frac{1}{b\left(\text{X}\right)}\left[{\dot{x}}_{nd}\left(t\right)-f\left(\text{X}\right)-d\left(t\right)-{\dot{e}}_{n}\left(t\right)+\dot{s}\left(t\right)+\lambda s\left(t\right)\right]$$

Here, *s*(t) represents a sliding surface constructed using two main state variables on the phase plane, *i.e.*,:

(4) $$s\left(t\right)=\left(\frac{d}{dt}+\lambda \right)e_{1}=e_{2}+\lambda e_{1}$$

Since it is usually very difficult to measure all state variables in a system with complicated dynamics, the sliding variable *s* will be adopted as the input signal in designing the fuzzy logic controller to approximate the so-called perfect control law, *u_eq_* (*t*). Substituting the perfect control law shown in Equation (3) into Equation (2), the following can be achieved:

(5) $$\dot{s}\left(t\right)+\lambda s\left(t\right)=0$$

**Figure 3.**
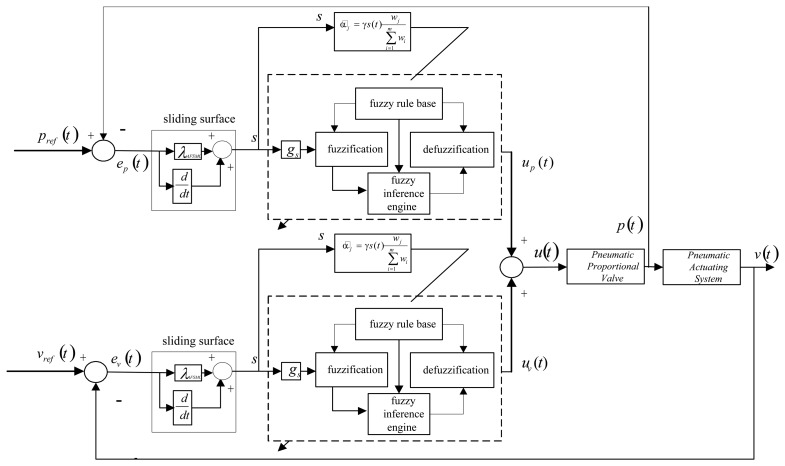
The overall control block diagram of the AFSMC control scheme.

**Figure 4.**
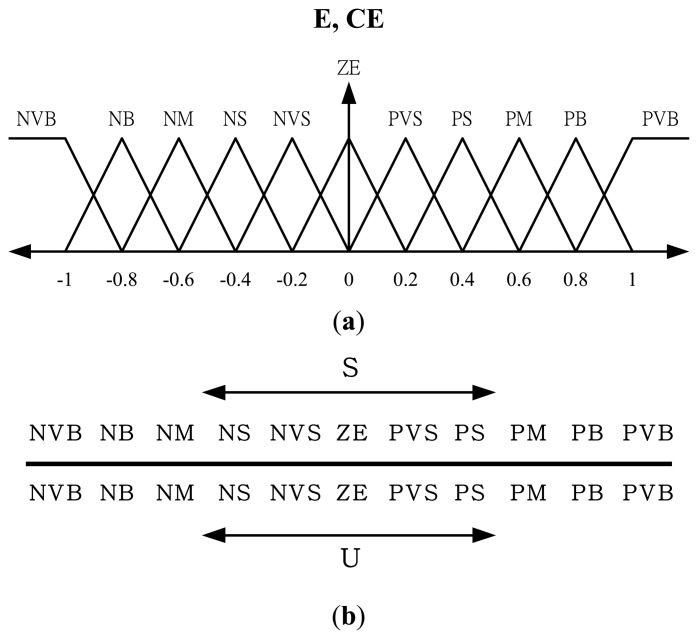
**(a)** Membership functions of the errors and error changes (Fuzzy partition with eleven terms: NVB, negative very big; NB, negative big; NM, negative medium; NS, negative small; NVS, negative very small; ZE, zero; PVS, positive very small; PS, positive small; PM, positive medium; PB, positive big; PVB, positive very big); **(b)** Fuzzy rules of AFSMC.

Equation (5) implies that the closed-loop system possesses asymptotical stability because *λ* is a positive constant. Thus, the sliding surface variable, *s*, will gradually converge to zero so does the control error, *e*_1_ . Note that in our control design scenario, two feedback signals, namely chamber pressure and payload velocity, are adopted to design their corresponding AFSMC. Since the design procedures of these two feedback loops are exactly the same, only the velocity feedback control case is taken for the illustrative purpose in the followings. To that end, we redefine the sliding surface variable as:

(6) $$s\left(t\right)=\left(\frac{d}{dt}+\lambda \right)e_{v}={\dot{e}}_{v}+\lambda e_{v}$$

where *e_v_* = *v_d_*(*t*)−*v*(*t*) representing the control error of payload velocity. In this paper, a fuzzy control scheme is employed to approximate the mapping between the sliding surface variable, *s*, and the control input, *u*. Thus, no model-based calculation is required. Although perfect control law may not be feasible in real situations, the error states can still converge to small error bound if proper control action is designed. In fact, the fuzzy logic control is employed to approximate the nonlinear control input, *u_eq_* (*t*) in this study. As shown in Figure 3, the proposed control strategy is a model-free FLC structure with a direct adaptive learning mechanism to adjust the fuzzy parameters based on sliding-mode stability criterion. Unlike the equivalent control input shown in Equation (3) which is derived from the nominal model at the sliding surface, the control voltage change for each sampling step here is derived from fuzzy inference and defuzzification calculation. Thus, the controller design can be accomplished without the knowledge of mathematical model. The input signal of this fuzzy control is the sliding surface variable, *s* , defined in Equation (6) which consists of the payload velocity and acceleration deviations. The one-dimensional (1-D) fuzzy rules shown in Figure 4(b) will be designed on the basis of the reaching condition for the sliding surface, namely *sṡ* < 0. The control law or control input generated from this velocity feedback loop is the output of the FLC. Based on the fuzzy inference decision and defuzzification operation, the control law can be represented as:

(7) $$u_{v}\left(t\right)=\frac{\overset{m}{\underset{i=1}{\text{∑}}}w_{i}U_{i}}{\overset{m}{\underset{i=1}{\text{∑}}}w_{i}}=\frac{\overset{m}{\underset{i=1}{\text{∑}}}w_{i}\alpha_{i}}{\overset{m}{\underset{i=1}{\text{∑}}}w_{i}}=\sum \phi_{i}\alpha_{i}$$

where *m* is the number of fuzzy rules and *w_i_* represents the firing weight of the z'th rule that has been activated. *U_i_* denotes the output from the z'th implication while *α_i_* is the associated consequent parameter which is adjustable. An online parameter tuning algorithm is proposed to adjust the consequent parameters for monitoring the system control performance. The adaptive rule is derived from the steep descent rule to decrease the value of *sṡ* with respect to *α_i_*. Thus, the updating law for the consequent parameter can be represented as:

(8) $${\dot{\alpha }}_{i}=-\Gamma \frac{\partial s\left(t\right)\dot{s}\left(t\right)}{\partial \alpha_{i}\left(t\right)}$$

in which Γ represents the learning gain. By means of chain rule, Equation (8) can be further rearranged into:

(9) $$\begin{array}{ll} {\dot{\alpha }}_{i}= & -\Gamma \frac{\partial s\left(t\right)\dot{s}\left(t\right)}{\partial u\left(t\right)}\frac{\partial u\left(t\right)}{\partial \alpha_{i}\left(t\right)}=\Gamma b\left(\text{X}\right)s\left(t\right)\frac{\partial u\left(t\right)}{\partial \alpha_{i}\left(t\right)}\\ & \equiv \gamma s\left(t\right)\frac{w_{i}}{\overset{m}{\underset{i=1}{\text{∑}}}w_{i}}=\gamma s\left(t\right)\Phi \end{array}$$

where the learning gain, Γ, and the system input parameter, *b*(**X**), are combined as a learning rate parameter, *γ *. Based on this on-line learning equation, the central positions of the defuzzification membership functions can be continuously regulated through the on-line modification of *α_i_*. This adaptive rule has two contributions. Firstly, it avoids the trial-and-error process in finding appropriate fuzzy rules. Secondly, it not only improves the closed-loop stability but also increases the convergent speed in driving the system states to sliding surface. The latter is true because steep descent rule has been applied in deriving the adaptive learning rule.

Now, since the proposed control scheme has on-line learning ability in adjusting fuzzy rules, the number of fuzzy rules is not as important as it is in traditional FLC [9]. In this paper, eleven fuzzy rules are employed to capture system dynamical response so as to obtain satisfactory control performance. The input membership functions are scaled into a range of −1 and +1 with equal span. To that end, a scaling factor *g_s_* is adopted to map the sliding surface variable, *s* into this universe of discourse. The membership function of fuzzy input variable and the fuzzy rules of the proposed AFSMC are illustrated in Figure 4(a,b), respectively.

Note that, since the fuzzy consequent parameter, *α_i_* , is updating continuously except for the conditions where *s*(*t*) is equal to zero, the consequent fuzzy parameter may drift and increase in one direction. In addition, the incessant small correction generated by the control scheme can cause the un-smoothness of control law and consequently the output oscillation. Hence, the modification [16] and dead-zone [17] concepts of nonlinear adaptive control are introduced in this fuzzy parameter correction equation to improve the smoothness and robustness of this AFSMC controller. To this end, the updating equation of the *i*th fuzzy consequent parameter (central value of membership function) can be described as:

(10) $${\dot{\alpha }}_{i}=\{\begin{array}{ll} 0 & ,if\;s(t)\leq d_{0}\\ \gamma s\left(t\right)\Phi -\kappa \left|s\left(t\right)\right|\alpha_{i}, & if\;s\left(t\right)>d_{0} \end{array}$$

where *κ* > 0 and *d*_0_ is a positive constant.

Note that, although the controller design described so far focuses on the velocity feedback loop, similar design procedures can be taken to handle the pressure-feedback control loop. To that end, the overall control block diagram can be obtained as in Figure 3.

## 4. Experimental Investigation

It is widely known that the performance of pneumatic vibration isolation system deteriorates as the excitation frequency gets close to the system's natural frequency. In order to show the enhancement of the isolation performance achieved by using the proposed active-isolation algorithms, sinusoidal excitation with the excitation frequency approximately equal to the system's natural frequency and a pseudo-random ground vibration were input through the floor base of the pneumatic-isolation system. Acceleration responses of the passive and active isolator were measured which were then numerically integrated to obtain velocity responses of the payload. Isolation performances obtained from the proposed AFSMC algorithm were compared with those of the passive isolator and the PID-active approach.

During experimental investigations, the sampling rate was taken as 1,000 Hz. The payload mass is 42 kg, which was supported by an isolation mount. The pressure of air supplied to the proportional control valve was 4.3 × 10^5^ Pa, whereas the static pressure in the chamber was 2.2 × 10^5^ Pa. The natural frequency of the experimental system is close to 3.7 Hz. As mentioned previously, two kinds of excitations were adopted to validate the proposed control algorithm. These include a 4 Hz sinusoidal ground vibration and a pseudo-random disturbance. These excitations were generated by an electromagnetic shaker. In addition, the active controllers were implemented using personal computer. Control parameters used in the AFSMC algorithm are listed in Table 1. The comparisons were performed using time-domain characteristics of the payload's velocity responses.

**Figure 5.**
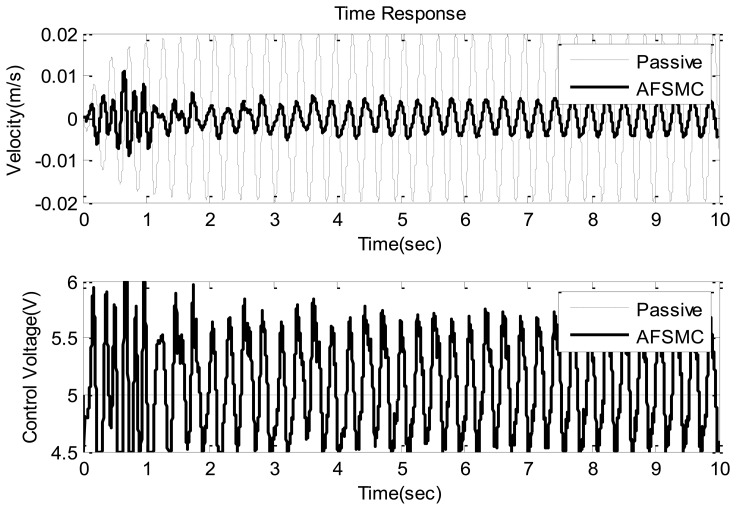
Time domain velocity response (upper) and control voltage (lower) of the passive and AFSMC-active isolator under sinusoidal disturbance.

**Figure 6.**
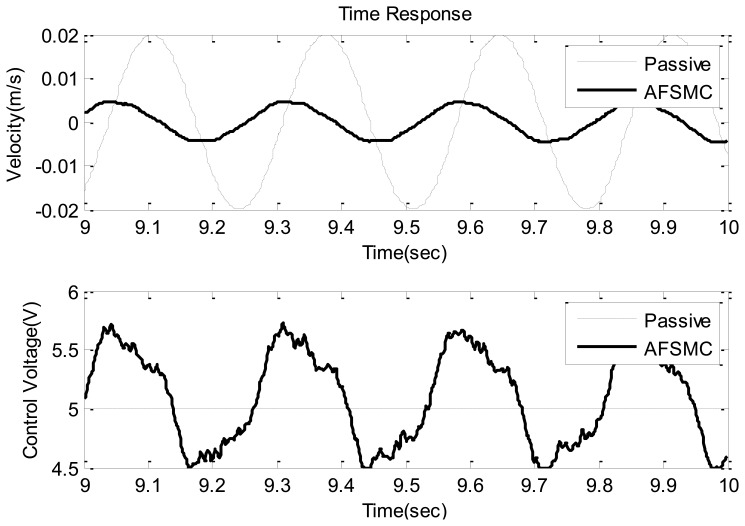
Time domain velocity response (upper) and control voltage (lower) of the passive and AFSMC-active isolator under sinusoidal disturbance (9−10 s).

**Figure 7.**
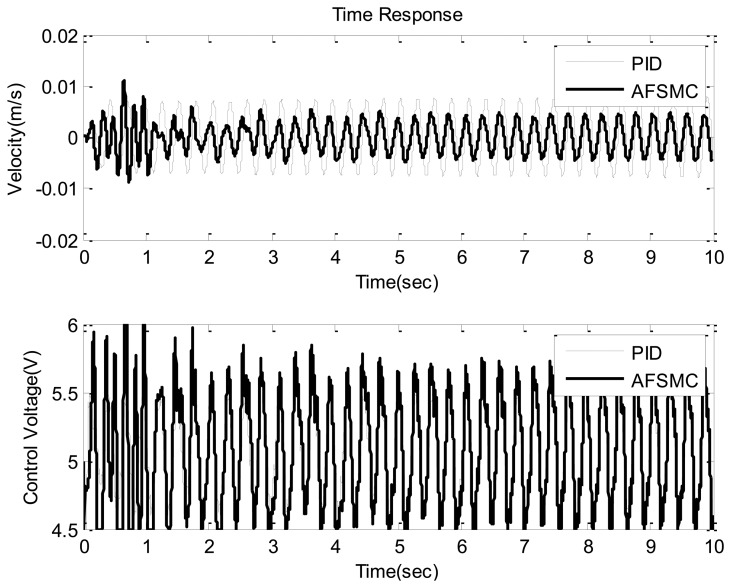
Time domain velocity response (upper) and control voltage (lower) of the AFSMC-active and PID-active (*k_p_*= 1,*k_t_*= 0.01,*k_d_* = 0.5) isolator under sinusoidal disturbance.

Typical time-domain responses including payload velocity and control voltage under sinusoidal ground vibration are presented in Figures 5 and 6 where Figure 5 shows the global features of the test while Figure 6 presents zoom-in details. In these plots, performance obtained using passive isolation is compared with that of the AFSMC-active isolation. It can be observed from these figures that the active isolators can significantly suppress the vibrations of the payload. In fact, a close examination reveals that the maximum velocity in the passive-isolation case was 0.02 m/s, whereas the largest velocity corresponding to the AFSMC-active isolators was 0.004 m/s. A reduction of 80% of the largest velocity amplitude was achieved by using the AFSMC approach. In addition, when the root mean square values (RMS) of the payload velocity are considered, the results are 0.0137 m/s and 0.003 m/s for the passive and AFSMC cases, respectively. The reduction of RMS value is about 78% in which the AFSMC again outperforms the passive approach. To further examine the effectiveness of the proposed control scheme, control performance obtained from the AFSMC-active case is compared with those obtained using traditional PID scheme. The results are presented in Figure 7.

Based on Figure 7, one can observe that although the reduction of payload velocity (from the PID approach to the AFSMC) is not as substantial as that shown in Figure 5, the AFSMC evidently outperforms the traditional PID scheme. The reduction of amplitude in this case is about 20%. Note that adopting the AFSMC over the traditional PID can not only provide better suppression performance but also avoid trial-and-error process required in selecting proper PID parameter values.

**Figure 8.**
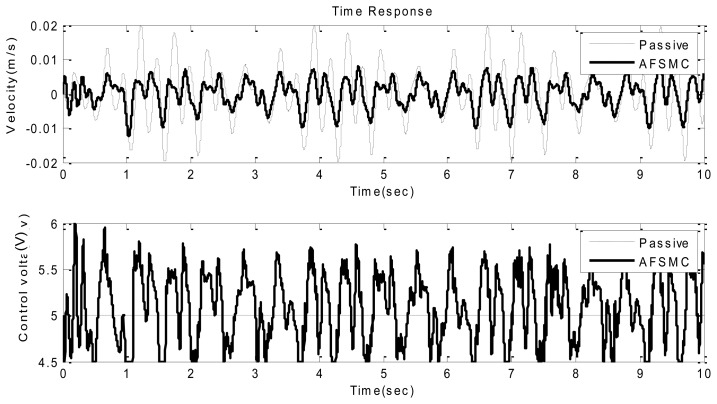
Time domain velocity response (upper) and control voltage (lower) of the passive and AFSMC-active isolator under random disturbance.

**Figure 9.**
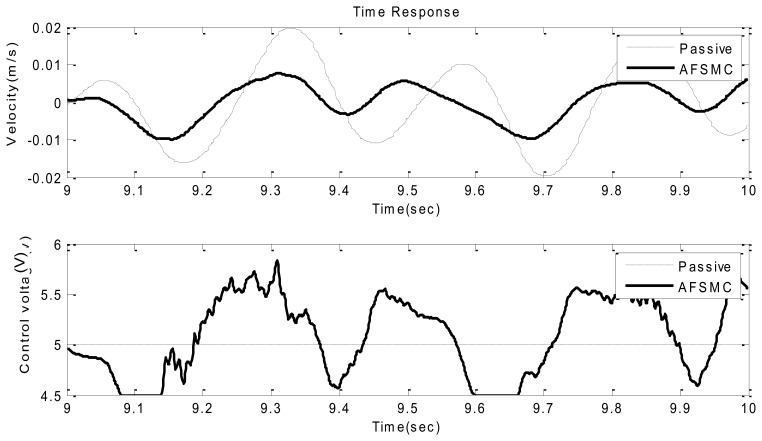
Time domain velocity response (upper) and control voltage (lower) of the passive and AFSMC-active isolator under random disturbance (9−10 s).

**Figure 10.**
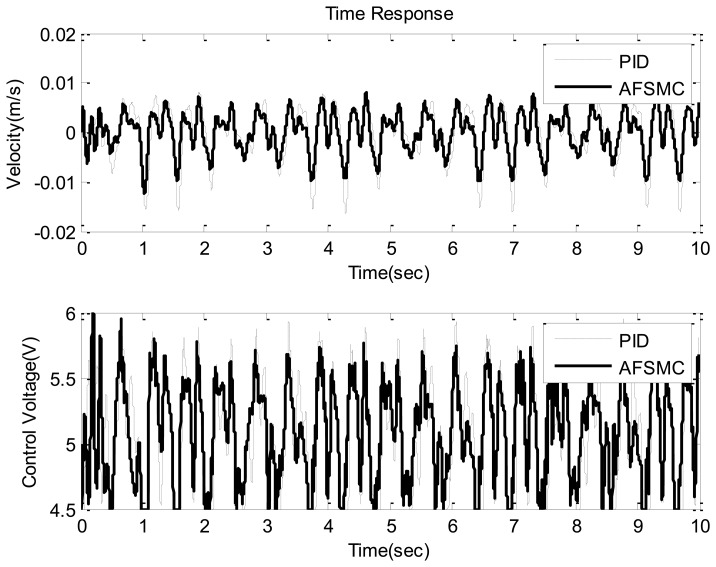
Time domain velocity response (upper) and control voltage (lower) of the AFSMC-active and PID-active (*k_p_*= 1,*k_i_* = 0.01,*k_d_* = 0.5) isolator under random disturbance.

Next, a random-like disturbance was adopted to further illustrate the suppression effectiveness of the proposed AFSMC scheme. As such, typical time-domain responses including payload velocity and control voltage input under random-like ground disturbance are presented in Figures 8 and 9. It can be observed from these figures that compared to passive approach, the proposed AFSMC scheme can significantly suppress the random-like vibrations of the payload. Since the input possesses random features, only the RMS values of the payload velocity are of concerned in this case. It turns out that the RMS values of the payload velocity are 0.009 m/s and 0.004 m/s for the passive and AFSMC-active isolation, respectively. The reduction is about 50% in this excitation case. Furthermore, a comparison between the AFSMC and PID approaches under random-like excitation is illustrated in Figure 10. It can be seen from Figure 10 that the control performance of the proposed AFSMC is better than that of the traditional PID controller. Although the reduction of payload velocity in this case seems not as evident as the results depicted in Figure 8 and 9, the AFSMC approach also saves the trial-and-error process required in determining the PID control parameters. Based on the experimental investigation conducted so far, one can conclude that the AFSMC active-isolation scheme works quite well in suppressing both sinusoidal disturbance at resonance and random-like disturbance.

## 5. Conclusions

In this paper, an active control methodology aiming to enhance the isolation performance of pneumatic isolators in low-frequency range is proposed. The approach applies adaptive fuzzy sliding-mode controller (AFSMC) to not only capture the nonlinear and time-varying system dynamics but also to counteract the payload response so that vibration suppression can be accomplished. The proposed AFSMC scheme combines an adaptive rule with fuzzy and sliding-mode control. Since the proposed approach is model-free, tremendous modeling effort regarding pneumatic isolation system can be avoided. The latter is known to be very challenging because many nonlinear phenomena are involved in such a pneumatic system. Experimental results have shown that the proposed AFSMC-active isolation approach can indeed suppress the vibration disturbance effectively. The study also demonstrates the feasibility of designing active isolation system using both payload velocity and chamber pressure as feedback signal. Moreover, based on experimental investigations, one can conclude that the performance of the proposed AFSMC scheme is better than that of the traditional PID controller. The AFSMC approach can also save the trial-and-error effort required in implementing the PID control strategy.

## Figures and Tables

**Table 1.** Values of control parameters used in AFSMC.

**Controller Type**	**Feedback Type**	**Parameter Values**
AFSMC	pressure	*g_s_* = 1; *g_u_* = 0.5; *λ* = 0.1; *γ* = 0.5
	velocity	*g_s_* = 1; *g_u_* = 15; *λ* = 0.1; *γ* = 0.5
